# The Impact of a Negative Media Event on Public Attitudes Towards Animal Welfare in the Red Meat Industry

**DOI:** 10.3390/ani10040619

**Published:** 2020-04-03

**Authors:** Maxine Rice, Lauren M. Hemsworth, Paul H. Hemsworth, Grahame J. Coleman

**Affiliations:** Animal Welfare Science Centre, Faculty of Veterinary and Agricultural Sciences, University of Melbourne, Parkville, VIC 3010, Australia; lauren.hemsworth@unimelb.edu.au (L.M.H.); phh@unimelb.edu.au (P.H.H.); grahame.coleman@unimelb.edu.au (G.J.C.)

**Keywords:** public attitudes, behaviour, media, live export, animal welfare, red meat industry

## Abstract

**Simple Summary:**

The public’s perception of a livestock industry can affect the sustainability of the industry. We used a random telephone survey of the Australian public to examine the effects on public attitudes of an Australian media campaign exposing animal cruelty in live export of sheep by sea. We found no apparent differences between those respondents that completed the survey before or after this media campaign in their concern for sheep or beef cattle welfare, their attitudes to red meat farming, acceptability of the red meat industry or their trust in farmers in the red meat industry. However, prior to the media campaign, respondents believed sheep to be more comfortable when transported by boats than those who completed the survey after the media campaign. While the media coverage was widespread, caution is required in the interpretation of these results as the survey questionnaire did not specifically ask if the respondents had seen the media coverage. Nevertheless, the coverage appears to have had little impact on public attitudes, either because the message itself had little impact or because of lack of exposure to the message.

**Abstract:**

Public perception of livestock industries and consumer trust in farmers can affect consumer behaviour and impact on social license to farm. Coincidental with a large random telephone survey of Australian public attitudes and behaviour towards the red meat industry, a media campaign exposing animal cruelty in live export of sheep by sea, occurred. Data collected from the nationwide survey of the public attitudes immediately before (n = 278 respondents) and after (n = 224 respondents) this media campaign was utilised in the present study to examine the effects of the media campaign on the public. In general, respondents’ attitudes towards the red meat industry were positive. Independent t-tests revealed no significant differences between those respondents that completed the survey before or after the 60 Minutes programme in their concern for sheep or beef cattle welfare, attitudes to red meat farming, acceptability of the red meat industry or their trust in farmers in the red meat industry. However, prior to the media campaign, respondents believed sheep to be more comfortable when transported by boats than did respondents who completed the survey after the media campaign. More respondents after the 60 Minutes programme cited social and internet media as a source of information. Therefore, despite the wide media coverage associated with the 60 Minutes programme, these results indicate little effect on the public’s attitudes towards farm animal welfare and the red meat industry. The significant impacts of the programme were reflected in increased community discussion, increased social media activity and an increase in the perceived importance of conditions aboard boats used for live sheep transport.

## 1. Introduction

In 2017 the total value of live exported sheep, goat and cattle was $1275 million AUD, making Australia one of the world’s largest exporters of live animals [1]. While the industry has clear economic benefits, concern for animal welfare largely in relation to the conditions on board ships and the treatment of animals abroad have led to several public media campaigns calling for bans to live export of animals [2]. Buddle and Bray [3] carried out a systematic analysis of the way in which the media frame farm animal welfare issues in Australia. They identified several themes that the media frequently addressed that were critical of the Australian livestock industries. These included live animal exports and free-range egg labelling. A significant theme of their argument was that such media coverage, in conjunction with active promotion of good animal welfare by retailers, provides a strong basis for increased ethical consumption and improved farm animal welfare.

Media coverage in relation to animal welfare has been shown to impact consumer demand for meat in the US [4]. In addition, media showing animal cruelty elicits emotional responses such as pity and hatred of the industry in some viewers [5], and so called “shock advocacy” commonly used in animal rights campaigns has been shown to negatively impact the credibility of the industry portrayed whilst at the same time increasing the credibility of the advocacy group behind the campaign [6].

Despite media coverage in relation to at least eight separate major incidents portraying poor animal welfare and animal cruelty between 2014 and 2016 [2], there has been no significant decline in Australia’s live export industry [7].

Social licence to farm, or the freedom within which society allows farmers to operate, is largely built on trust within the community [8]. As the welfare of animals, particularly in the livestock industries, is becoming an increasing societal concern [9,10], public perception of the industry and consumer trust in farmers, which is shown to affect consumer behaviour [11], will have an increasing impact on social licence to farm. But how much impact do media campaigns in relation to specific aspects within the red meat industry have on the public’s attitude towards red meat production in general?

This paper investigated the impacts of a large media campaign exposing animal cruelty in live export of sheep on public attitudes and behaviour towards the Australian red meat industry, as assessed by a nationwide survey of the public which, by coincidence, was collecting data immediately prior to and after the event. Footage portraying animal cruelty filmed by a whistle-blower aboard the Australian live sheep export ship Awassi Express was aired on the Australian television current affairs programme 60 Minutes [12]. The footage, in particular, refers to the high temperature and humidity on board the ship and shows animals displaying signs of heat stress (panting). Subsequent to the programme, for several days there was wide print media coverage, talk-back radio coverage, news reporting and increased social media activity. Sinclair, et al. [13] evaluated public reactions to this event using a short questionnaire, collecting responses from 522 respondents, approached at random in Brisbane, Australia. They found that 71% of respondents were familiar with the live export trade before the media coverage and this increased to 78.5% following the coverage. However, feelings of positivity (or negativity) towards the live export trade did not change. 

It is not known whether any other public attitudes or behaviour in regard to the live export trade or the livestock industries in general were impacted by this media coverage. Media coverage of adverse events in the livestock industries tends to include graphic footage, increased talkback radio activity and increased social media activity. Such coverage provides one of the infrequent opportunities for the general public to see first-hand, some aspects of the livestock industries. It is not known what the immediate effects of such exposure might be. The aim of this paper is to investigate broader changes in the community than those studied by Sinclair et al. [13].

## 2. Materials and Methods 

### 2.1. Development and Structure of Questionnaire

The survey was developed using an iterative process beginning with questionnaires that had been developed by the Animal Welfare Science Centre (AWSC) for a range of livestock industries including the pork, egg and red meat industries (see [14,15,16]). These questionnaires were consolidated and modified to target attitudes towards the red meat industry and animal welfare in relation to specific issues in the red meat industries. The questionnaires also assessed the participant’s knowledge of farm animals and farm animal welfare, the frequency with which they accessed information on farm animal welfare, and the source of information they most frequently used and trusted. Topics covered in the questionnaire are outlined in Table 1.

### 2.2. Recruitment of Participants and Data Collection

Human ethics approval was obtained from The University of Melbourne’s Human Ethics Advisory Group. I-view, a specialised market and social research data collection agency, were contracted to deliver the questionnaires to the general public using random telephone recruitment (Computer Assisted Telephone Interviewing — CATI) of 500 participants. 

CATI involved dialling random fixed-line and mobile telephone numbers Australia-wide and inviting potential participants to complete the questionnaire by phone. In each call, the consultant requested the youngest male in the household as preference in order to counteract the expected bias for both older, and/or female participants.

Australia-wide data collection for CATI commenced on 21 March 2018 and was completed on the 16 April 2018. The average duration of the CATI survey was 36 mins. Initially only landline numbers were used, however despite asking for the youngest male (to try to reduce the tendency for females to respond first) in the household, there was still a considerable bias towards older females. In order to counteract this bias, I-view began including mobile numbers and targeted areas with more youthful demographics from 26 March onwards.

The 60 Minutes programme portraying animal cruelty was aired during the period of data collection, on the 8 April, 2018. Details of the specific voyage are available in the corresponding Mortality Investigation report 69 [17]. On the 1 August 2017, a total of 63,804 sheep and 50 cattle were exported by sea from Fremantle destined for ports in Qatar, Kuwait and United Arab Emirates. During the 23 day voyage the ship recorded severe high temperatures with some decks reaching 37 °C (wet bulb temperature), high humidity and heat affected sheep over four consecutive days which resulted in boggy deck conditions, and ultimately a reported mortality rate of 3.76% [17]. The current *Australian Standards for the Export of Livestock Version 2.3* [18] identifies shipboard mortality rates of equal to or greater than 2% in sheep to be a notifiable incident which must be reported to the relevant Australian government agency as soon as possible and within 12 hours.

On the night the footage was aired, the television current affairs programme 60 Minutes ranked 6 in the national Free-to-Air programme ratings with an estimated 630,000 viewers [19]. Programmes that ranked higher included the commonwealth games on 3 separate channels, and 2 news programmes. The documentary sparked over 20 related online news articles and 2 public protests in the fortnight that followed (Source: Factiva Global News Database).

### 2.3. Data Analysis

Using IBM SPSS statistics for windows (version 26), a series of independent t-tests were conducted comparing the responses from the survey collected prior to the news event, and those collected post the news event across a range of variables. There was almost an even spread of responses across the two periods (n = 278 pre-event (21 March–7 April 2018), n = 224 post-event (9 April–16 April 2018). Given the large sample size, the validity of the assumption of normality of the distribution of the variables for the t test was considered irrelevant [20]. Equal variances were not assumed.

To summarise some of the responses to multifaceted questions, composite scores were developed. Attitude and trust questionnaire data were analysed using Principal Components Analysis (PCA) followed by either a Varimax or an Oblimin rotation to identify commonalities amongst the questionnaire items. Items that were established as belonging to a common underlying component were then summed to produce a composite score for that component. Before conducting the PCAs, items were recoded where appropriate so that high scores reflected positive attitudes, high trust etc. The reliability of these calculated scores was measured using Cronbach’s Alpha. 

## 3. Results

### 3.1. Participant Demographics

Table 2 shows the final breakdown of the age/gender demographics from the Australia-wide CATI survey both pre and post the 60 Minutes Programme, despite a bias for female participants prior to the 60 Minute programme, there was good representation of male and female participants in all age groups both prior to and post the programme.

The geographical distribution of participants both pre and post the 60 Minutes programme is presented in Table 3.

### 3.2. Principle Component Analysis

A summary of the composite scores, their components and Cronbach’s alpha coefficients is outlined in Table 4. The majority of the composite scores had a Cronbach’s Alpha greater than 0.7 and were considered reliable. Two of the composite scores “Caring for and balancing the needs of pets and people” and “Easy to act” had coefficients less than 0.7 (0.57 and 0.48 respectively), while these were not considered reliable, they were retained because the component groupings showed good face validity and only comprised two items which tends to reduce the magnitude of Cronbach’s Alpha.

### 3.3. Independent T-test Analyses of Participant Responses pre and post the 60 Minutes Programme

A series of independent t-tests were conducted comparing the responses from the survey collected prior to the news event, and those collected post the news event across a range of variables. The data from the CATI survey had almost an even spread of responses across the two periods (n = 278 pre-event, n = 224 post-event).

There was no significant difference in the eating habits of respondents pre and post the 60 Minutes Programme in relation to their consumption of meat and regularity of meat consumption (Table 5).

Results of the independent t-tests comparing the composite score variables of respondent’s pre and post the 60 minutes programme are presented in Table 6. In general, respondents’ attitudes towards the red meat industry were positive. Based on the independent t-tests there were no significant differences between those respondents that completed the survey before or after the 60 Minutes programme in their meaning of animal welfare, concern about sheep (mean score = 2.73 out of 5, where 1 means “extremely concerned” and 5 means “not concerned”) or beef cattle welfare (mean score = 2.70 out of 5), or the acceptability of the animal uses (mean score = 3.0 out of 5, where 1 means “strongly disapprove” and 5 means “strongly approve”). In addition, there was no significant difference between the two groups of respondents in their behavioural, normative or control beliefs perceived and actual knowledge (in relation to livestock practices) or attitudes towards most red meat farming practices (including their approval of farming practices, their beliefs around importance of farming attributes) or their trust in farmers in the red meat industry. However, prior to the news event, participants completing the survey believed sheep to be more comfortable when transported by boats than those who completed the survey post the news event (Table 6).

While the survey did not distinguish between passively or actively accessing information, significantly more respondents cited social and internet media as a source of information after the 60 Minutes programme than those completing the questionnaire prior to the programme based on the composite scores; however, there was no significant difference between the two groups of respondents in their use of any other source of information, or in their trust of the different information sources (Table 7).

Respondents completing the questionnaire after the 60 Minutes programme indicated that they had told more people about farm animal welfare than those that completed it before the programme, however they were not more likely to be asked about farm animal welfare or be used as a source of advice on farm animal welfare (Table 8).

## 4. Discussion

On the basis of these results, it is clear that, despite the wide media coverage that the 60 Minutes programme received, there was little effect on the public’s attitudes towards the red meat industry. In fact, the significant impacts of the programme were reflected in increased community discussion and increased social media activity and an increase in the perceived importance of conditions aboard boats used for live sheep transport. Respondents’ underlying attitudes and beliefs about farm animal welfare were not affected.

Adverse media events occur frequently, they receive considerable publicity and often there is industry or government reactions [2]. However, it is not known whether the effect of these media events is as significant or pervasive as that believed by industry or regulators. The government reaction to an earlier exposé on animal mistreatment in Indonesian abattoirs, “A Bloody Business” by Four Corners on Australian television [21], was to ban live export to Indonesia temporarily which resulted in significant industry impacts and poor welfare outcomes for cattle held in feedlots for a significant period of time [22]. Thus, this wide coverage resulted in a government reaction which itself received wide industry condemnation and substantial political debate. This political response was in the absence of data on the nature or extent of public concern. The point here is that media coverage of these events, even if they do not have major impact on the population, can have significant political consequences.

In this current research, there was an increase in social and internet media activity reported by respondents post the media event. It is possible that this increased activity merely reflected the overall amount of information available on social media rather than increased engagement by the public. However, the mere fact that overall activity increased suggests that people became more actively engaged. Furthermore, the question in the questionnaire used in this study asked people how regularly they had obtained information from social media. Although respondents were not asked if they were members of a specific social media group, it can reasonably be assumed that most members of the general public are not members of animal welfare-specific groups and therefore would need to actively choose to read such information. This is reinforced by the finding of Buddle et al. [23] who found little to no cross-over between animal activists and industry on social media. Previous research by Tiplady et al. [5], which surveyed the public post-media coverage of animal cruelty, showed very similar results in that, while the news event sparked more discussions and a self-reported determination to take action, very few respondents performed any activities that would have an impact on the industry in question.

While individual media events may not have a significant immediate impact on public attitudes, there may be an incremental effect over time. That is, if you get adverse event after adverse event the cumulative effect may change the public’s views, and this is supported to some extent by Tonsor and Olynk [4] who reported a small but significant impact of animal welfare-related media on consumer demand for pork and beef. However, it does appear that a single adverse event, at least in this instance, does not have a significant effect on broader public attitudes towards the livestock industries. It is possible that this occurs because people become desensitised to livestock animal welfare issues. However, there is anecdotal evidence of media campaigns and associated public reactions having a significant impact, over time, on government policy. A good example of this is the recent “Me Too” movement on sexual harassment. Given that Australian citizens are not the end consumer of the live export products, it is also possible that there is a disconnect between their attitudes towards the red meat industry in general and their attitudes towards the live export industry, and a more detailed survey specifically targeting attitudes towards live export would be required.

The kind of engagement that people had in terms of communication, not specifically for or against any particular issue, also changed after the 60 Minutes programme. People became more engaged with the topic of farm animal welfare in the period after, thus the media event achieved the goal of generating more animal welfare-related discussions. However, the source of the information communicated in these discussions needs to be considered. The increase in social and internet media activity suggests that Facebook and Twitter activity increased dramatically around that time. Similar increases in social media activity were reported by Buddle et al. [23] who examined the impact of a wool campaign run by People for the Ethical Treatment of Animals (PETA) exposing the mistreatment of sheep in Australian shearing sheds. This is perhaps not surprising because social media activity is an immediate reaction facilitated by the accessibility of social networking sites through smart phones and personal media devices. This does suggest that, as a communication medium between producers and consumers, social media might be a good option, however Buddle et al. [23] also reported little to no cross-over between the domains of the animal activists (i.e., followers of animal activist groups on social media) and that of the industry (i.e., followers of agricultural groups) on topics supporting agricultural industry, suggesting that these discussions are limited to their respective micro-publics Thus, challenges remain in ensuring that both parties are represented in the discussions. 

Some caution must be used in the interpretation of these results as the questionnaire did not specifically ask if the respondents had seen the media coverage, so it is possible that a fair proportion had not seen the coverage, although the changes in communication discussed previously do not support this. Be that as it may, given the wide coverage that the 60 Minutes programme received, the random methodology of the participant recruitment and the large sample size it is reasonable to assume that a large proportion of the respondents had received some exposure to the media event. Nevertheless, the coverage appears to have had little impact, either because the message itself had little impact or because of lack of exposure to the message.

## Figures and Tables

**Table 1:** Structure of the questionnaire.

Section	Information Gathered
A. Demographics	Age, gender, education, location, red meat consumption,
B. Animal welfare	General attitudes towards animal welfare, normative and control beliefs in relation to animal welfare
C. Knowledge of farm animals and farm animal welfare	Perceived and actual knowledge of beef cattle and sheep production practices (e.g., curfew, mulesing and castration)
D. Attitudes towards red meat farming practices	Approval of red meat farming practices, importance of social contact, fresh air, exercise etc., concern about transport conditions.
E. Behaviour in relation to farm animal welfare	Animal rights group membership, activities to express dissatisfaction with sheep and beef cattle farming, sources of animal welfare information, discussions about animal welfare

**Table 2:** Age/gender demographics (by percentage %) of CATI survey prior to (pre) and after (post) the 60 Minutes Programme (Census data in italics where available).

	Pre (%)			Post (%)			Overall (%)		
Age	Male	Female	Other	Male	Female	Other	Male	Female	Other
18–24	28	72		52	48		40	60	
25–34	45	55		49	51		47 *(49)*	53 *(51)*	
35–44	55	45		52	48		54 *(49)*	46 *(51)*	
45–54	27	73		50	50		39 *(49)*	61 *(51)*	
55–64	52	48		55	45		53 *(49)*	47 *(51)*	
65+	34	64	2	53	47		43 *(46)*	56 *(54)*	1
Overall	40	60	0	52	48		46 *(49)*	54 *(51)*	1

**Table 3:** Geographic location of participants in the CATI survey pre and post the 60 Minutes programme.

		Count		Census (% by State)
		Pre	Post	
**Location-State**	Melbourne	63	40	24
	Rest of Victoria	24	10	
	Sydney	34	39	29
	Rest of New South Wales	30	34	
	Brisbane	27	25	22
	Rest of Queensland	34	23	
	Adelaide	21	10	8
	Rest of South Australia	8	2	
	Perth	9	28	12
	Rest of Western Australia	10	3	
	Hobart	5	1	3
	Rest of Tasmania	5	3	
	Australian Capital Territory	7	3	2
	Northern Territory	1	3	1

**Table 4:** Components from the questionnaire grouped into composite scores, a high score indicates positive attitude or strong agreement to the statements (questionnaire items). Cronbach’s Alpha was calculated using the full sample.

Topic	Attitude Component	Cronbach’s Alpha	Questionnaire Item
The meaning of animal welfare	Humane treatment	0.82	Humane treatment of animals
			Preventing animal cruelty
			Protecting the rights of animals
	Best practice handling	0.78	Farmers and farm animal handlers using best practice
			Farmers and farm animal handlers caring for their animals
	Caring for and balancing the needs of pets and people	0.57	Caring for our pets
			Balancing the needs of animals and people
Acceptability of animal uses	Red meat attributes	0.81	I believe beef and lamb are healthy foods
			It is appropriate to use sheep and beef cattle to produce food for humans
			Sheep and beef cattle farming is environmentally sustainable
			Sheep and beef cattle are raised in a humane and animal friendly manner
	Red meat animal rights	0.69	Sheep and beef cattle have the same right to life as domestic animals
			Sheep and beef cattle have the same feelings as domestic animals
Behavioural beliefs	Public engagement beliefs	0.89	I think it is important to lobby governments to improve the welfare of farm animals
			I should encourage my friends to support animal welfare causes
			It is important for me to be actively involved in the promotion of farm animal welfare
			It is important for me to encourage family and friends to be actively involved in the promotion of animal welfare
Normative beliefs	Negative normative beliefs	0.74	The welfare of farm animals is not something that my partner/family would expect me to consider when making meat shopping choices
			Lobbying the government to improve the welfare of farm animals is not something my partner/family would expect me to do
			My partner/family would not expect me to encourage my family and friends to be actively involved in the promotion of animal welfare
	Positive normative beliefs	0.78	My partner/family would expect me to buy lamb and beef that is produced with good animal welfare practices
			My partner/family would expect me to encourage my friends to support animal welfare causes
			My partner/family would expect me to be actively involved in the promotion of farm animal welfare
Control beliefs	Difficult to act	0.48	I find it takes too much effort to buy beef and lamb that is produced with good animal welfare practices.
			I would find it too difficult to lobby the government to improve the welfare of farm animals
	Easy to act	0.75	I can easily encourage my friends to support animal welfare causes
			I can easily be involved actively in the promotion of farm animal welfare
Trust of livestock industry people	Trust	0.92	I trust farmers to properly care for their sheep and beef cattle
			I trust farm animal handlers to properly care for their sheep and beef cattle
			I trust those responsible for transporting sheep and beef cattle by land to properly care for them
			I trust abattoir workers who work with sheep and beef cattle to properly care for them and use humane slaughter methods
Attitudes towards red meat farming practices	Approval of husbandry practices	0.89	Mulesing
			Crutching
			Dehorning
			Pre-slaughter stunning
			Curfew
			Tail docking
			Ear tagging
			Hot iron branding
			Castration
			Feedlotting
			Spaying
Importance of farming attributes	General welfare	0.95	Social contact with animals of the same species
			Contact with their young
			Shelter
			Access to water
			Freedom to roam outdoors
			Good nutrition
			Regular exercise
			Fresh air
			Protection from predators
			Pain relief during painful husbandry procedures
	Medication	0.8	Medications (i.e., antibiotics) for health
			Vaccinations for health
Comfort of beef cattle	Land beef transport conditions	0.94	Space per animal
			Provision of food and water
			Ventilation
			Journey length
			Road/truck conditions (e.g., sound, vibration, braking levels
			Loading of animals onto vehicles (e.g., use of handling aids, human handling)
	Sea beef transport conditions	0.96	Space per animal
			Provision of food and water
			Ventilation
			Journey length
			Boat conditions (e.g., sounds, vibration, unsteady ground)
			Loading of animals onto boats (e.g., use of handling aids, human handling)
Comfort of sheep	Land sheep transport conditions	0.96	Space per animal
			Provision of food and water
			Ventilation
			Journey length
			Road/truck conditions (e.g., sound, vibration, braking levels
			Loading of animals onto vehicles (e.g., use of handling aids, human handling)
	Sea sheep transport conditions	0.97	Space per animal
			Provision of food and water
			Ventilation
			Journey length
			Boat conditions (e.g., sounds, vibration, unsteady ground)
			Loading of animals onto boats (e.g., use of handling aids, human handling)
Accessing information	Commercial media	0.79	Government advertisements/promotions
			Celebrity chef/cook
			Industry bodies
			Supermarkets (e.g., Coles, Woolworths, IGA)
			Labels (product labels)
	Social and internet media	0.8	Internet
			Friends, relatives or colleagues
			Animal welfare organizations e.g., RSPCA
			Social network sites, related social media (e.g., Facebook, YouTube, Twitter, blogs)
	Conventional media	0.75	Television (e.g., TV news, documentaries)
			Radio
			Print media (e.g., magazines, newspapers, scientific papers)
Trust of information sources	Trust social and internet media	0.84	Television (e.g., TV news, documentaries)
			Radio
			Internet
			Print media (e.g., magazines, newspapers, scientific papers)
			Friends, relatives or colleagues
			Animal welfare organizations e.g., RSPCA
			Social network sites, related social media (e.g., Facebook, YouTube, Twitter, blogs)
	Trust conventional media	0.82	Government advertisements/promotions
			Industry bodies
			Supermarkets (e.g., Coles, Woolworths, IGA)
			Labels (product labels)
			Celebrity chef/cook

**Table 5:** Independent t-tests comparing the eating habits of respondents completing the survey prior to vs post the 60 Minutes Programme.

Eating Habits	t	df	Significance 2-Tailed	Mean		Mean Difference	Standard Error Difference
				Pre	Post		
Eats meat	0.69	500	0.49	1.10	1.12	0.02	0.03
How often would you eat beef in an average week?	0.73	500	0.47	3.26	3.34	0.08	0.11
How often would you eat lamb in an average week?	0.91	500	0.36	2.36	2.44	0.08	0.09

**Table 6:** Independent t-tests comparing the beliefs of the respondents pre and post the 60 Minutes programme in relation to the question “What does animal welfare mean to you?”. Responses based on composite scores.

Topic	Variable	t	df	Significance 2-Tailed	Mean		Mean Difference	Standard Error Difference
					Pre	Post		
Meaning of animal Welfare	Humane treatment	0.16	500	0.87	4.41	4.42	0.01	0.08
	Best practice handling	−0.16	500	0.88	4.29	4.27	−0.01	0.08
	Caring for and balancing the needs of pets and people	−1.10	500	0.27	4.11	4.01	−0.09	0.09
Concern for	Sheep welfare	−1.73	500	0.09	2.66	2.46	−0.20	0.11
	Beef cattle welfare	−1.39	500	0.17	2.61	2.45	−0.17	0.12
Acceptability of animal uses	Red meat attributes	0.12	500	0.91	3.64	3.65	0.01	0.09
	Red meat animal rights	1.19	500	0.24	3.94	1.05	0.11	0.09
Behavioural, normative and control beliefs	Public engagement beliefs	0.51	500	0.61	3.49	3.54	0.05	0.10
	Negative normative beliefs	0.11	500	0.91	2.88	2.89	0.01	0.10
	Positive normative beliefs	−0.58	500	0.57	3.32	3.27	−0.06	0.10
	Difficult to act	0.60	500	0.55	3.05	3.04	−0.06	0.09
	Easy to act	0.35	500	0.73	3.11	3.15	−0.04	0.10
Perceived knowledge	Beef cattle production	−0.49	500	0.62	2.83	2.78	−0.05	0.10
	Sheep production	−0.44	500	0.66	2.96	2.91	−0.05	0.11
Actual knowledge	Knowledge Score	0.89	500	0.37	71.64	73.08	1.44	1.61
Attitudes towards red meat farming practices	Approval of husbandry practices	1.06	500	0.29	3.00	3.09	0.09	0.08
Importance of farming attributes	General welfare	−0.82	500	0.41	4.78	4.75	−0.03	0.03
	Medication	−1.14	500	0.25	4.58	4.51	−0.07	0.07
Comfort of beef cattle	Land beef transport conditions	−0.65	500	0.52	2.53	2.46	−0.07	0.10
	Sea beef transport conditions	−1.71	500	0.09	2.18	2.01	−0.16	0.10
Comfort of sheep	Land sheep transport conditions	−1.34	500	0.18	2.41	2.28	−0.14	0.10
	Sea sheep transport conditions	−2.15	500	0.03	2.12	1.91	−0.21	0.10
Trust of farmers	Trust	−1.30	500	0.19	3.43	3.29	−0.14	0.10

**Table 7:** Independent t tests comparing the perceived sources of information and the trust of information sources of respondents pre and post the 60 Minutes programme (based on composite scores).

Source of Information	t	df	Significance 2-Tailed	Mean		Mean Difference	Standard Error Difference
				Pre	Post		
Commercial media	0.32	500	0.75	2.01	2.03	0.02	0.06
Social and internet media	2.45	500	0.02	2.64	2.86	0.21	0.09
Conventional media	1.59	500	0.11	2.55	2.68	0.13	0.08
Trust commercial media	−1.07	500	0.29	2.65	2.57	−0.08	0.07
Trust social and internet media	−0.18	500	0.86	2.97	2.96	−0.01	0.07
Trust conventional media	1.19	500	0.23	2.98	3.08	0.10	0.08

**Table 8:** Independent t tests comparing the communication activities that respondents engaged in pre and post the 60 Minutes programme.

Communication Activities	t	df	Significance 2-Tailed	Mean		Mean Difference	Standard Error Difference
				Pre	Post		
During the past six months, how many people have you told about farm animal welfare in Australia?	2.54	500	0.01	2.19	2.52	0.33	0.13
Compared with your friends, how likely are you to be asked about farm animal welfare in Australia?	1.52	500	0.13	2.17	2.35	0.18	0.12
Overall, in all of your discussions with friends and neighbours how often are you used as a source of advice on farm animal welfare in Australia?	0.85	500	0.40	1.84	1.92	0.08	0.10
